# Hypo-osmolar rectal douche tenofovir formulation prevents simian/human immunodeficiency virus acquisition in macaques

**DOI:** 10.1172/jci.insight.161577

**Published:** 2022-12-08

**Authors:** Peng Xiao, Sanjeev Gumber, Mark A. Marzinke, Thuy Hoang, Rohan Myers, Abhijit A. Date, Justin Hanes, Laura M. Ensign, Lin Wang, Lisa C. Rohan, Richard Cone, Edward J. Fuchs, Craig W. Hendrix, Francois Villinger

**Affiliations:** 1 New Iberia Research Center, University of Louisiana at Lafayette, New Iberia, Louisiana, USA.; 2 Division of Pathology, Yerkes National Primate Research Center, Emory University, Atlanta, Georgia, USA.; 3 Division of Clinical Pharmacology, Department of Medicine;; 4 Department of Pathology;; 5 Center for Nanomedicine; and; 6 Department of Pharmacology and Molecular Sciences, Johns Hopkins University School of Medicine, Baltimore, Maryland, USA.; 7 Department of Pharmacology and Toxicology, R. Ken Coit College of Pharmacy, University of Arizona, Tucson, Arizona, USA.; 8 Department of Ophthalmology and Vision Science, University of Arizona, Tucson, Arizona, USA.; 9 Department of Biomedical Engineering, Johns Hopkins University, Baltimore, Maryland, USA.; 10 Department of Ophthalmology, Johns Hopkins University School of Medicine, Baltimore, Maryland, USA.; 11 Department of Chemical & Biomolecular Engineering, Johns Hopkins Whiting School of Engineering, Baltimore, Maryland, USA.; 12 Department of Pharmaceutical Sciences, School of Pharmacy, University of Pittsburgh, Pittsburgh, Pennsylvania, USA.; 13 Department of Obstetrics, Gynecology, and Reproductive Sciences, University of Pittsburgh, Pittsburgh, Pennsylvania, USA.; 14 Magee-Womens Research Institute, Pittsburgh, Pennsylvania, USA.

**Keywords:** AIDS/HIV, Virology, Pharmacology

## Abstract

In spite of the rollout of oral pre-exposure prophylaxis (PrEP), the rate of new HIV infections remains a major health crisis. In the United States, new infections occur predominantly in men having sex with men (MSM) in rural settings where access to PrEP can be limited. As an alternative congruent with MSM sexual behavior, we have optimized and tested tenofovir (TFV) and analog-based iso-osmolar and hypo-osmolar (HOsm) rectal douches for efficacy against rectal simian/human immunodeficiency virus (SHIV) infection of macaques. Single TFV HOsm high-dose douches achieved peak plasma TFV levels similar to daily oral PrEP, while other formulations yielded lower concentrations. Rectal tissue TFV-diphosphate (TFV-DP) concentrations at the portal of virus entry, however, were markedly higher after HOsm douching than daily oral PrEP. Repeated douches led to significantly higher plasma TFV and higher TFV-DP concentrations in rectal tissue at 24 hours compared with single douches, without detectable mucosal or systemic toxicity. Using stringent repeated intrarectal SHIV exposures, single HOsm high-dose douches delivered greater protection from virus acquisition for more than 24 hours compared with oral PrEP. Our results demonstrate a rapid delivery of protective TFV doses to the rectal portal of virus entry as a potential low-cost and safe PrEP alternative.

## Introduction

An estimated 1.5 million new human immunodeficiency virus (HIV) infections were reported worldwide in 2020 (1), attesting to only a slow decline in the transmission rate of HIV. Even in industrialized countries, new infections still represent a significant public health burden. However, the front of new infections in the United States has largely shifted to the South in men who have sex with men (MSM), which account for 70% of all new HIV infections (2). The risk of colorectal HIV transmission after unprotected receptive anal intercourse (RAI) is 10- to 20-fold higher than after vaginal transmission (3). In addition, US MSM reported not using condoms in their last sexual encounter 38%–65% of the time (4, 5). In certain areas, the incidence rates among African American MSM are similar to those in regions of HIV-endemic sub-Saharan Africa or Asia (5–7). Therefore, high RAI frequency, low condom use, and higher risk of new HIV infection represent a perfect storm that calls for multiple strategies to prevent HIV infection in this group. Preexposure prophylaxis (PrEP) with oral daily tenofovir (TFV), as either the prodrug TFV disoproxil fumarate (TDF) or TFV alafenamide (TAF), in fixed-dose combination with emtricitabine (FTC) achieves excellent protection (8–13) in MSM and transgender women (TGW), but the rollout of PrEP has been slow for rural MSM and TGW for several reasons, and a recent report shows a gradual decrease of use over time associated with increased infection (14). Barriers to PrEP uptake and adherence include concerns regarding discrimination or perceptions regarding HIV status and sexual practices. Even when these barriers are overcome, strict adherence to the treatment can be difficult due to access to the clinical center (distance and limited mobility), out-of-pocket costs, and potential side effects even though new drugs have been better tolerated. Thus, there is a need to provide alternative options that are easier to manage and will limit side effects associated with daily dosing (10, 15–21). A preventive measure used prior to or just after RAI could address some of the limitations of oral PrEP, such as the Ipergay trial designed to test a pericoital oral dosage of antiretroviral therapy (22), and long-acting injection PrEP trials to compare with daily oral PrEP (23, 24). Moreover, previous trials have shown that to be effective, preventive measures are far easier to establish if they are embedded into ongoing pericoital practices. Rectal douching with enemas is very common among MSM, with up to 88% of men who practiced RAI ever douching for cleansing purposes and 43%–64% reporting recent douching (25). Typically, douches are used within 2 hours before and 1 hour after sex (albeit less often) for reasons of hygiene, pleasure enhancement, and false belief in HIV protection (26–28). Thus, taking advantage of this practice to deliver rectal microbicides was reasoned to lead to a high likelihood of adherence (28, 29).

Topical delivery of TFV has been investigated for vaginal delivery to prevent HIV acquisition with different outcomes. CAPRISA 004, iPrEx, TDF2, Partners PrEP, VOICE, and FEM-PrEP trials have been conducted using TFV gel (30), though failures have often been traced back to poor adherence. Rectal delivery has also been investigated in the form of gels for safety and acceptability (31–36) but not as a douche or tested for efficacy. We recently demonstrated that TFV hypo-osmolar (HOsm) douching in rhesus monkeys markedly promotes local drug uptake and faster enzymatic turnover to TFV-diphosphate (TFV-DP) in colorectal tissues compared with iso-osmolar (IOsm) douche formulations, and this delivery yields durable protection against SIV and simian/human immunodeficiency virus (SHIV) infection ex vivo using sequentially collected rectal explants (37). In the present study, we also tested various other TFV-based and TFV prodrug–based (TAF and CMX157) IOsm and HOsm formulations by single-dose pharmacokinetics (PK) studies and in rapid sequences of doses using the HOsm douche formulation with the best PK. Based on these data, we conducted a final proof-of-efficacy study using repeated intrarectal SHIV challenges of monkeys pretreated with a single topical douche in comparison with daily oral PrEP regimens adjusted to mimic blood and tissue human drug exposures of TDF and FTC in standard oral PrEP regimens.

## Results

### PK.

**Figure 1:**
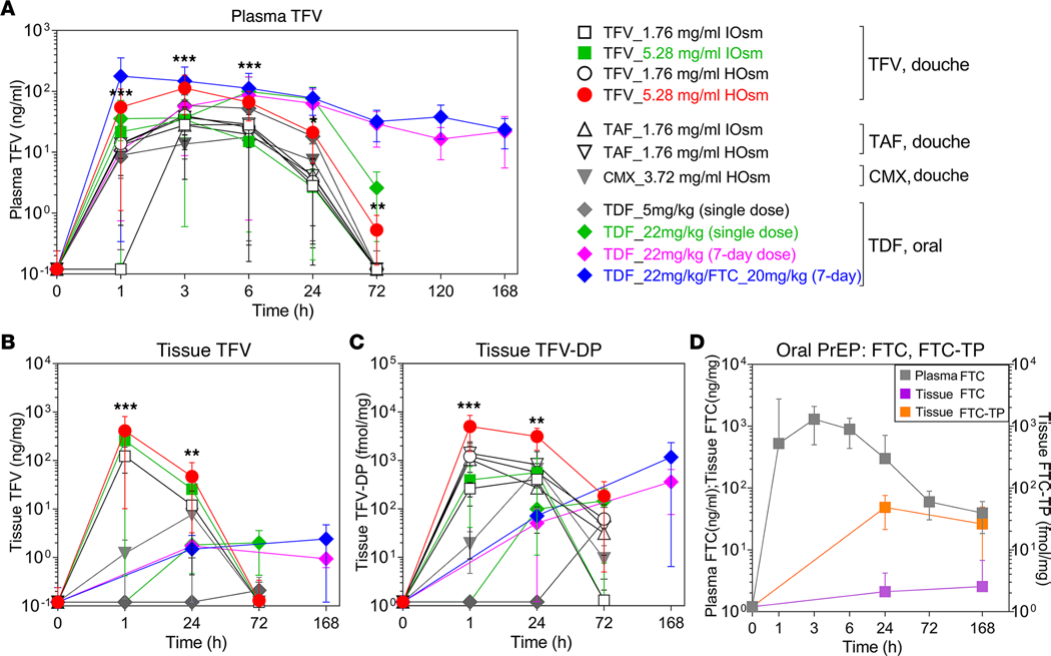
PK profile of TFV/TFV prodrug IOsm and HOsm douche formulations and oral TDF and TDF/FTC regimens in rhesus macaques. Median values with 95% CI of (**A**) plasma TFV, (**B**) colorectal tissue TFV, and (**C**) tissue TFV-DP concentrations after administration in groups of 6 monkeys each. (**D**) Median plasma FTC, colorectal tissue FTC, or tissue FTC-TP after 1-week daily oral PrEP (22 mg/kg TDF combined with 20 mg/kg FTC). One-way ANOVA was used to compare TFV 5.28 mg/mL HOsm to all other douche formulations. The level of significance is indicated by *P* values as follows: *P < 0.05; **P < 0.01; ***P < 0.001. PK, pharmacokinetic; HOsm, hypo-osmolar; IOsm, iso-osmolar; TFV, tenofovir; TFV-DP, tenofovir diphosphate; TDF, tenofovir disoproxil fumarate; FTC, emtricitabine; FTC-TP, emtricitabine triphosphate.

IOsm and HOsm rectal douche formulations of TFV and TFV prodrugs (TAF and CMX157) were evaluated for plasma TFV and tissue TFV-DP after single-dose administration PK studies and compared with oral single and repeated daily-dose PrEP regimens to assess the systemic and colorectal tissue drug concentrations delivered by each formulation (Figure 1). Plasma TFV concentrations tended to peak at 3 hours for most douches and oral administrations. A notable exception among the douche formulations was the CMX HOsm formulation, which peaked at 6 hours (Figure 1A and Table 1). Of interest, among the oral administrations, the TDF/FTC combo also showed an earlier peak of plasma TFV concentrations compared with TDF administration as a single agent. All single-dose administrations gradually declined to low or unquantifiable (<0.31 ng/mL) by 72 hours.

Among the douche formulations, the TFV HOsm high dose (5.28 mg/mL) achieved earlier plasma TFV concentrations with significantly higher C_max_ (103 ng/mL, *P* < 0.001, Table 1) and consistently higher median plasma TFV concentrations and plasma AUC_24h_ (1,247 ng×h/mL; average 3.5-fold, *P* < 0.001) when compared with all other douche formulations (Table 1, Figure 1A, and Supplemental Figure 2A; supplemental material available online with this article; https://doi.org/10.1172/jci.insight.161577DS1). This HOsm douche formulation also achieved TFV concentrations comparable to oral PrEP regimens at 1, 3, and 6 hours (Supplemental Figure 2A) and plasma AUC_24h_ values similar (*P* = 0.148) to oral PrEP regimens (Table 1). The high-dose HOsm douche formulation resulted in an AUC_last_ value close to the steady-state AUC reported in humans receiving a once-daily oral dose of 300 mg of TDF (2,300 ng×h/mL) (38, 39). No statistical differences were noted among plasma TFV C_max_ (*P* = 0.567), AUC_24h_ (*P* = 0.549), or AUC_last_ (*P* = 0.558), respectively, following low-dose douching with TFV or TAF (1.76 mg/mL) or CMX-based (3.72 mg/mL) douching, irrespective of the osmolarity (Table 1). Contrary to our expectations, CMX157 douching did not result in extended TFV release in plasma. The single 22 mg/kg TDF dose showed comparable plasma C_max_, T_max_, and AUC_24h_ results to the daily repeated 22 mg/kg TDF (Table 1). Of interest was the observation that the combination of FTC (20 mg/kg) with TDF (22 mg/kg) was associated with increased absorption of TDF compared with daily oral TDF (22 mg/kg) alone, as indicated by an earlier TFV peak concentration (T_max_, 1 hour), higher C_max_ (177 ng/mL), and significantly higher steady-state AUC_last_ (8,872 ng×h/mL, *P* = 0.014) (Table 1 and Table 2). In addition, FTC/TDF combination induced a synergistic effect on FTC activity as well (Figure 1D) and resulted in a higher AUC_24h_ value (19,987 ng×h/mL, Table 1 and Table 2) than results reported for monkeys treated with oral 20 mg/kg FTC alone (13,200 ng×h/mL) (39) and humans receiving 200 mg FTC (13,100 ng×h/mL) (40).

Following a single-dose rectal douche, the TFV high-dose HOsm formulation yielded the highest TFV concentrations in tissues (410 ng/mg, Figure 1B) and significantly higher TFV-DP concentrations in colorectal tissues collected at 1 hour (*P* < 0.001) and 24 hours (*P* < 0.01), respectively, when compared with other rectal formulations and oral PrEP regimens (Figure 1C and Supplemental Figure 2, B and C). These TFV-DP values were 11.5-fold (*P* = 0.001) and 7.5-fold (*P* = 0.002) higher at 1 and 24 hours, respectively, after a single dose when compared with concentrations achieved at steady state after 7 daily oral 22 mg/kg TDF doses (Supplemental Figure 2, B and C). Similarly, these TFV-DP concentrations were 4.0-fold (*P* = 0.022) and 2.1-fold (*P* = 0.041) higher at 1 and 24 hours, respectively, compared with steady state (day 7) achieved by 7 daily oral doses of 22 mg/kg TDF with 20 mg/kg FTC (Supplemental Figure 2, B and C). The HOsm high-dose formulation also resulted in significantly higher tissue TFV-DP AUC values when compared with all other douche formulations (AUC_24h_, average 5.7-fold, *P* = 0.002; AUC_last_, average 4.5-fold, *P* = 0.005, respectively) (Table 1 and Table 2) or compared with the 2 oral PrEP regimens (AUC_24h_, average 128.1-fold, *P* < 0.001; AUC_last_, average 2.9-fold, *P* = 0.011, respectively).

Substituting TAF for TFV did not appreciably affect the tissue TFV-DP AUC values after a single low dose in IOsm and HOsm formulations (Table 1). In contrast, the CMX157 HOsm formulation resulted in significantly lower tissue TFV-DP AUC values when compared with the low-dose, TFV-based HOsm formulation (AUC_24h_, *P* = 0.002; AUC_last_, *P* = 0.026, respectively) or TAF-based, low-dose HOsm formulation (AUC_24h_, *P* = 0.004; AUC_last_, *P* = 0.026, respectively). CMX157 did not result in extended TFV or TFV-DP PK in tissues.

Tissue FTC and FTC-TP concentrations were also evaluated in the context of an oral PrEP regimen of 22 mg/kg TDF and 20 mg/kg FTC (Figure 1D). Although tissue FTC concentrations were quite low, relatively higher concentrations of tissue FTC-TP were achieved and maintained at steady state on day 7 (Figure 1D and Table 2).

Given the rapid and extensive uptake of TFV via rectal douching, it was important to investigate whether this uptake affected intracellular uptake and metabolism as well as potential toxicities. We evaluated whether any of the formulations would affect circulating blood cell populations by flow cytometry. Frequencies and activation levels of T cells, B cells, and NK cells did not show any detectable alteration at 24 and 72 hours after dosing (data not shown). Tissue histomorphologic analyses upon single-dose administration showed no evidence of mucosal damage at 24 hours and 72 hours relative to baseline (data not shown).

In addition to measuring TFV-DP in whole colorectal tissue homogenates, we also isolated mucosal CD4^+^ T cells from colorectal biopsies collected at 1 hour after douche administration. The TFV HOsm high-dose formulation induced markedly higher TFV-DP levels in mucosal CD4^+^ T cells compared with the same dose IOsm formulation as well as TAF-based IOsm and HOsm formulations (Supplemental Figure 2D).

### Rapidly repeated douche PK and safety.

**Figure 2:**
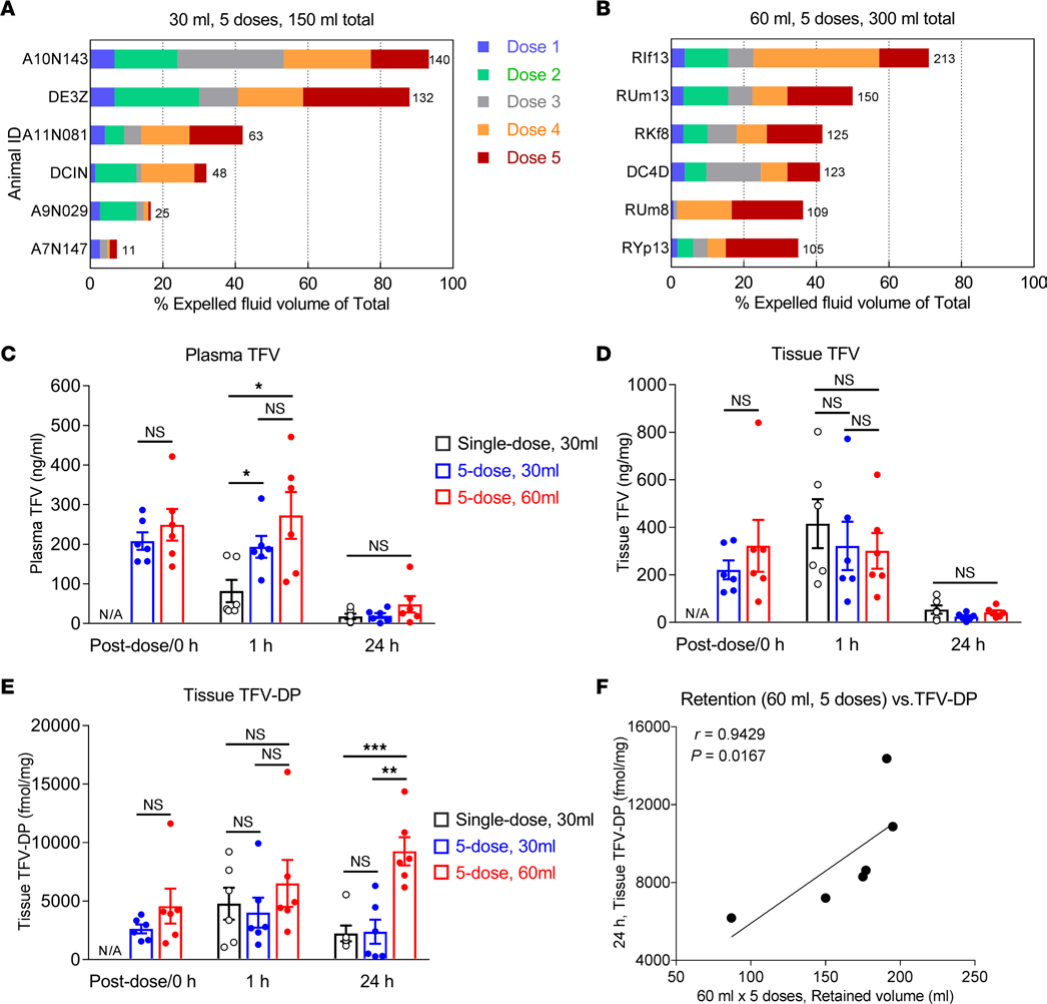
PK evaluation of 5 repeated short-term intrarectal douches of TFV HOsm high-dose (5.28 mg/mL) formulation. Expelled fluid volumes after 5 consecutive intrarectal administrations of (**A**) 30 mL and (**B**) 60 mL of HOsm high-dose formulation. (**C**) Comparison of plasma TFV between single-dose PK and 5 repeated doses PK. (**D**) Comparison of colorectal tissue TFV between single dose PK and 5 repeated doses PK. (**E**) Comparison of tissue TFV-DP between single-dose PK and 5 repeated doses PK. Differences between the 2 groups (6 monkeys each) were evaluated by the Wilcoxon rank-sum test. (**F**) PK correlation of retained volumes and TFV-DP concentration in rectal tissues. The correlation coefficients (*r*) and *P* values were derived using Spearman rank analysis. The level of significance is indicated by *P* values as follows: *P < 0.05; **P < 0.01; ***P < 0.001. PK, pharmacokinetic; HOsm, hypo-osmolar; TFV, tenofovir; TFV-DP, tenofovir diphosphate.

We studied repeated douches to test both the TFV PK and safety of a more clinically relevant multiple-douche sequence (25, 41–45). We subjected monkeys to 5 consecutive high-dose TFV HOsm rectal douches at 5-minute intervals, each of 30 mL followed by pressing fluid out abdominally before administering the next douche. As shown in Figure 2A, the median percentage of the 30 mL (5 times) douche volume administered (150 mL) that was expelled was 37% (range 7% to 90%) with less than half of the total volume expelled in 4 of 6 animals. When we doubled the douche volume to 60 mL 5 times, a larger percentage of the administered douche volume was expelled: median 41%, range 35% to 71% (*P* = 0.031, Figure 2B). However, the residual volume retained was larger in absolute terms: 30 mL (5 times) median 94 mL (range 10–139 mL), 60 mL (5 times) median 176 mL (range 87–195 mL) (*P* = 0.017).

Following repeated 30 mL and 60 mL doses of the HOsm high-dose formulation, among the 3 times observed, immediately postdose/0 hour, 1 hour, and 24 hours, there were generally no differences in TFV concentration in plasma or tissues among douche volume and number (Figure 2, C and D). There were significantly higher plasma TFV concentrations 1 hour after repeat dosing when compared with the single dose (30 mL 5 times, 4.4-fold, *P* = 0.015; 60 mL 5 times, 6.7-fold, *P* = 0.026) (Figure 2C) and significantly higher tissue TFV-DP concentrations 24 hours after 60 mL (5 times) dosing when compared with single and multiple 30 mL douches (*P* < 0.01 and *P* < 0.001, respectively) (Figure 2E). Correlation analyses of retained volumes and tissue TFV-DP concentrations exhibited a progressively positive trend but no significance over time after five 30 mL repeated doses (Supplemental Figure 3, A–C). Similarly, a progressively positive trend was observed after the five 60 mL repeated doses (Supplemental Figure 3, D and E), until the *P* value became significant at 24 hours (Figure 2F and Supplemental Figure 3F, *r* = 0.9429, *P* = 0.0167), suggesting potentially much higher and extended drug levels in tissues, given the increased amount of fluid retained.

### Mucosal and systemic tolerability of repeated short-term douches of HOsm high-dose TFV formulation.

**Figure 3:**
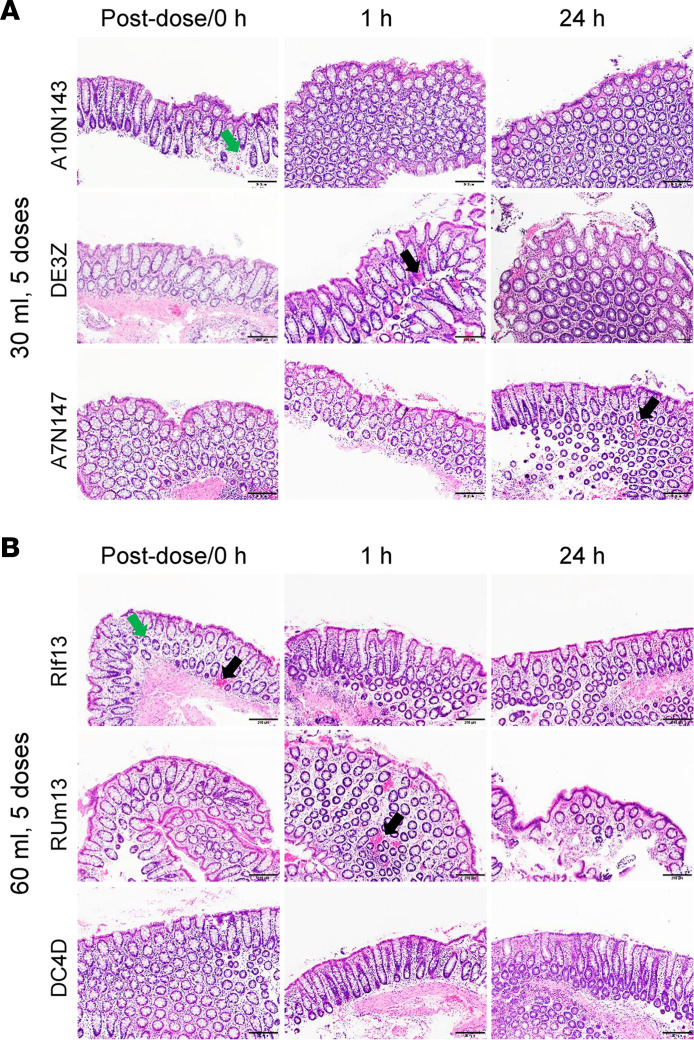
Mucosal toxicity testing of 5 repeated short-term intrarectal douches of TFV HOsm high-dose (5.28 mg/mL) formulation. Representative colorectal biopsy histomorphological photomicrographs from 3 animals at postdose (0 hour), 1 hour, and 24 hours after 5 consecutive intrarectal administrations of (**A**) 30 mL and (**B**) 60 mL of HOsm high-dose formulation. There was no evidence of mucosal damage at any time point in any animal; however, microhemorrhages (black arrows) and mild edema (green arrows) are indicated. Scale bar: 200 μm. HOsm, hypo-osmolar; TFV, tenofovir.

To address the question of tissue toxicity, tissue collections were conducted at the completion of the 5 douches, and at 1 and 24 hours after the repeated 30 mL or 60 mL doses of TFV HOsm high-dose douches, for histopathological analyses. Hematoxylin and eosin–stained colorectal tissue sections collected 24 hours after repeated dose volumes showed no mucosal damage at any time point in any animal except for microhemorrhages and edema that were routinely observed before dosing and appeared to be collection artifacts (Figure 3). There were also no noticeable changes in complete blood counts and serum chemistry panels (including electrolytes, liver enzymes, albumin, globulin, etc.) at baseline and 24 and 72 hours after the repeated 30 mL or 60 mL doses of TFV HOsm douches (data not shown). While these evaluations may not be considered fully comprehensive, they were not suggestive of any tissue or systemic alteration secondary to HOsm high-dose TFV douching.

### Ex vivo tissue explant SIV/SHIV challenge.

Using an ex vivo rectal explant challenge model of sequentially collected colorectal biopsies, we confirmed that the HOsm high-dose (5.28 mg/mL) formulation was highly effective in preventing SHIV/SIV infection (37). The same TFV dose formulated as IOsm douche or TAF at a lower dose (1.56 mg/mL), either in IOsm or in HOsm formulation, were less effective in preventing either SHIV162p3 (Supplemental Figure 4A) or SIVmac251 (Supplemental Figure 4B) virus replication ex vivo. The limited solubility of TAF did not allow for direct comparison with the high-dose TFV.

We also investigated TFV-DP in multiple draining lymph nodes (LNs) including colonic LNs draining the rectal mucosa after douching focusing on 3 and 24 hours. As shown in Supplemental Figure 2E, the TFV-DP concentrations were clearly quantifiable in all draining LNs at 3 hours after TFV HOsm high-dose douche administration when compared with the same IOsm formulation whereas spleen and select axillary LNs were below the threshold of detection at that time. By 24 hours postdosing, TFV-DP concentrations had increased to similar levels in both IOsm and HOsm formulations, irrespective of anatomical location (Supplemental Figure 2E).

### Efficacy study design and repeated intrarectal SHIV challenge outcome.

**Figure 4:**
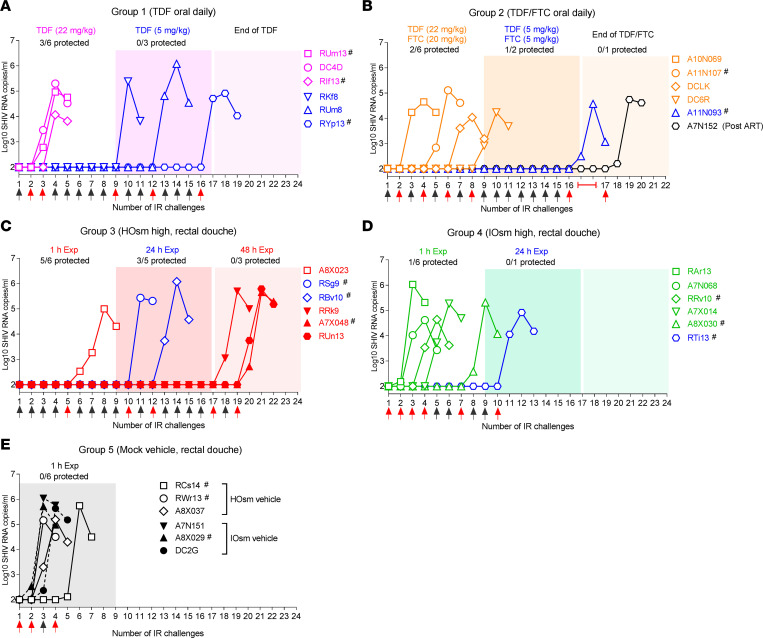
Outcome of efficacy study of weekly repeated intrarectal SHIV challenges. Plasma peak acute viral loads illustrate infection results following weekly repeated intrarectal challenges with simian/human immunodeficiency virus 162p3 (SHIV162p3) for (**A**) group 1, TDF oral daily; (**B**) group 2, TDF/FTC oral daily; (**C**) group 3, HOsm high; (**D**) group 4, IOsm high; and (**E**) group 5, mock vehicle control. Red arrow indicates time of infection and black arrows mark sequential weekly rectal SHIV challenges. # indicates Mamu A01^+^ macaque distribution among the groups. HOsm, hypo-osmolar; IOsm, iso-osmolar; TDF, tenofovir disoproxil fumarate; TFV, tenofovir; FTC, emtricitabine.

Since the TFV HOsm high-dose (5.28 mg/mL) douche formulation showed highly promising PK and explant challenge results, we moved this and control approaches to an in vivo efficacy model. As outlined in Supplemental Figure 1, 5 intervention groups (*n* = 6 per group) were established, including a mock douche control (group 5), and 2 reference groups of daily oral TDF 22 mg/mL PrEP (beginning 2 doses prior to the first viral challenge, group 1) and daily oral TDF 22 mg/kg (and FTC 20 mg/kg; group 2, equivalent to human dosing based on AUC), beginning 8 daily doses prior to the first rectal viral challenge to establish steady-state conditions. These 3 groups were compared with single-weekly rectal douche with TFV HOsm 5.28 mg/mL (group 3) and TFV IOsm 5.28 mg/mL (group 4). A series of 8 consecutive weekly rectal viral challenges were delivered 1 hour after the daily oral or weekly rectal dose. Subsequently, animals that resisted all 8 challenges were challenged for an additional 8-week period at 24 hours after the weekly rectal doses for groups 3 and 4. In this period, the daily oral doses were reduced to TDF 5 mg/kg (group 1) and TDF 5 mg/kg and FTC 5 mg/kg (group 2). The rectal douche groups (group 4 and group 5) continued for a third 8-week period in which uninfected animals from the prior 2 periods received weekly rectal viral challenges 48 hours after the weekly rectal douche. PrEP stopped for each animal once viremia was observed. In the first 8-week challenge period, group 5 control monkeys (3 HOsm and 3 IOsm vehicle only) were all rapidly infected: 2 monkeys after the first exposure, 3 after the second, and 1 after the fourth, attesting to the robust nature of the challenge (Figure 4E). Surprisingly, 3 of the 6 macaques in group 1 (oral daily TDF PrEP, 22 mg/kg) also acquired virus with the second and third rectal exposures (Figure 4A); the remaining 3 monkeys in this group became infected when the daily TDF dose was reduced to 5 mg/kg in the second 8-week period. Similarly, 4 of the 6 macaques in group 2 (oral daily PrEP with TDF 22 mg/kg and FTC 20 mg/kg) became infected after 8 or fewer exposures (range, 2–8, Figure 4B); an additional animal acquired the virus in the second 8-week period while treated with the lower daily dose of TDF/FTC, and the last animal was finally infected once PrEP had been discontinued. Five of 6 group 4 animals, challenged at 1 hour after TFV IOsm douche, became infected during the initial 8 challenges; the last animal became infected when the time interval between douche and viral exposure increased to 24 hours (Figure 4D). In contrast, the greatest protection (5/6 protected) was seen in group 3, dosed with the TFV HOsm douche 1 hour prior to SHIV exposures (Figure 4C) (*P* = 0.004, when compared with all other groups). When the time interval between douche and exposure was extended to 24 hours, only 2 of 5 remaining animals acquired the virus, while the other 3 animals finally became infected when exposed 48 hours after the TFV HOsm douche (Figure 4C).

Although the virus was clearly sensitive to TFV and FTC (Supplemental Figure 5), the peak viral loads among all infected animal groups were only diminished in group 2 (oral TDF/FTC) compared with all others (median SHIV RNA copies/mL in group 1, 5.2 log; group 2, 4.6 log; group 3, 5.7 log; group 4, 5.1 log; and group 5, 5.7 log; *P* = 0.011 relative to all other groups; Supplemental Figure 5F). Differences in viral kinetics were, however, noted during chronic infection (Supplemental Figure 5, A–E and G), whereby the PrEP groups exerted rapid control of the challenge even though further PrEP had been discontinued as soon as infection was confirmed. Animals in the IOsm or HOsm TFV groups also induced rapid control of SHIV, while control animals did not (*P* < 0.0001, Supplemental Figure 5G).

**Figure 5:**
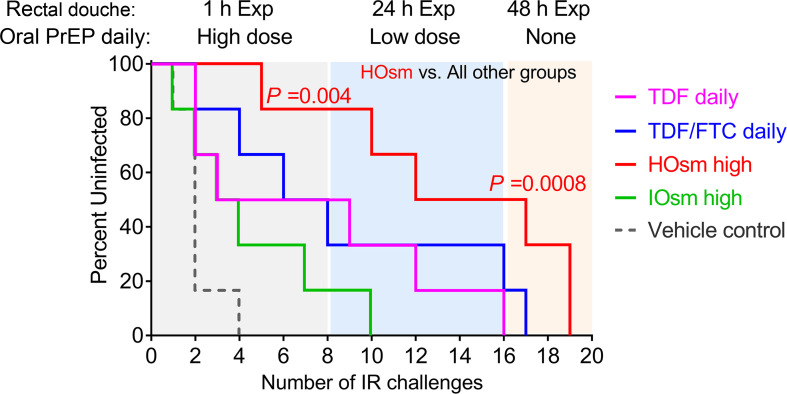
Protective efficacy of each intervention group against repeated intrarectal SHIV162p3 challenges. Number of challenges required for acquisition of infection in each group. Each Kaplan-Meier survival curve represents the cumulative percentage of uninfected macaques as a function of the number of weekly rectal virus exposures. Animals in the HOsm and IOsm groups were administered a single douche 1, 24, or 48 hours prior to challenge, while oral treatments were continuous. The exact log-rank test was used to ascertain statistical difference between groups. Statistical analyses were considered significant of *P* < 0.05. Exp, exposure to SHIV162p3; HOsm, hypo-osmolar; IOsm, iso-osmolar; TDF, tenofovir disoproxil fumarate; FTC, emtricitabine.

The protective efficacy of TFV HOsm high-dose formulation was compared with all other interventions: a Kaplan-Meier curve of virus acquisition was derived based on the number of exposures needed to achieve infection (Figure 5). Considering only the initial 8 challenges across all groups, the HOsm significantly reduced risk of acquisition compared with either daily oral TDF alone (*P* < 0.01) or daily oral PrEP TDF/FTC (*P* < 0.05). Overall, the difference between the HOsm and all other groups was highly significant (*P* = 0.004, Figure 5). Taking into consideration the entire challenge study, all controls became infected after a median of 2 rectal exposures (range, 1–4), and all 6 macaques in the IOsm group (green line) were infected after a median of 4 rectal exposures (range, 1–10). Macaques pretreated with daily oral TDF alone (pink line) were infected after a median of 6 rectal exposures (range, 2–16), which was similar to those pretreated with daily oral PrEP TDF/FTC (blue line, median of 7 rectal exposures, range, 2–17). In contrast, all monkeys pretreated with HOsm douche (red line) were infected after a median of 15 rectal exposures (range, 5–19), which significantly delayed SHIV acquisition compared to all other groups (*P* = 0.0008, Figure 5).

Relative to vehicle control, daily oral TDF alone had a 75% per-exposure acquisition risk reduction (Cox HR: 0.247, *P* = 0.0348; log-rank test, *P* = 0.0449), whereas the daily oral TDF/FTC PrEP resulted in 82% risk reduction (Cox HR: 0.176, *P* = 0.0109; log-rank test, *P* = 0.0087) (Table 3). In contrast, HOsm douche formulation afforded the highest risk reduction of 93% compared with vehicle control (Cox HR: 0.067, *P* = 0.0005; log-rank test, *P* = 0.0006). The protection afforded by the HOsm douche was greater than that of daily oral TDF alone (log-rank test, *P* = 0.0022), daily oral TDF/FTC group (log-rank test, *P* = 0.0101), and the IOsm group (log-rank test, *P* = 0.0061), respectively (Table 3).

### PK and pharmacodynamics correlation analysis for PrEP failures.

**Figure 6:**
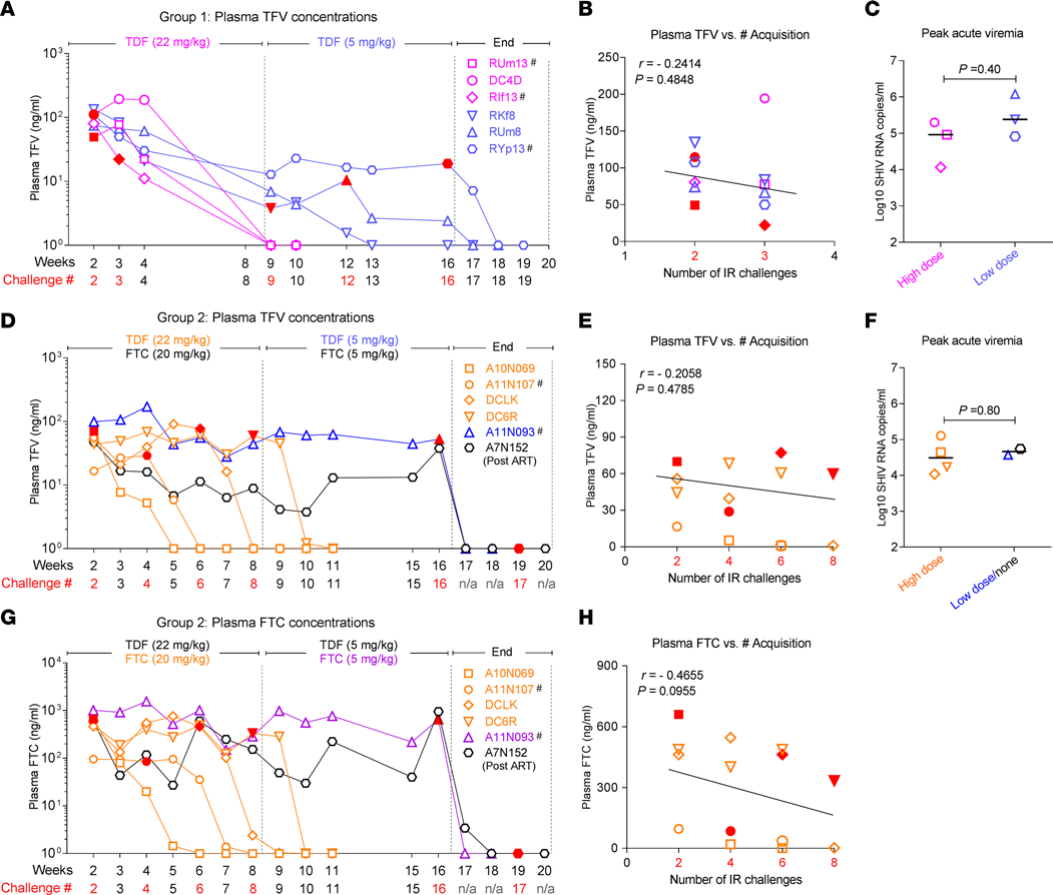
No correlations between virus acquisition and plasma drug levels in group 1 (TDF oral daily) or 2 (TDF/FTC oral daily). (**A**) Group 1 individual macaque plasma TFV value at time of infection (red-filled symbol) during oral TDF high-dose and low-dose treatment. # indicates Mamu A01^+^ macaques. (**B**) No correlation between virus acquisition and plasma TFV levels during high-dose TDF treatment in group 1. (**C**) Comparison of peak acute viremia of animals infected in high-dose or low-dose TDF treatment in group 1. (**D**) Group 2 individual macaque plasma TFV value at time of infection (red-filled symbol) during oral TDF/FTC high-dose and low-dose treatment. (**E**) No correlation between virus acquisition and plasma TFV levels during high-dose TDF/FTC treatment in group 2. (**F**) Comparison of peak acute viremia of animals infected in high-dose or low-dose and no TDF/FTC treatment in group 2. (**G**) Group 2 individual plasma FTC value at time of infection (red-filled symbol) during oral TDF/FTC high dose and low dose treatment. (**H**) No correlation between virus acquisition and plasma FTC levels during high-dose TDF/FTC treatment in group 2. The red number indicates time of infection. The correlation coefficients (*r*) and *P* values are from Spearman rank analysis. Differences between the 2 groups were evaluated by the Wilcoxon rank-sum test. Statistical analyses were considered significant for *P* values of < 0.05. TFV, tenofovir; TDF, tenofovir disoproxil fumarate; FTC, emtricitabine.

Considering the high protective efficacy of PrEP in the clinical setting, we next investigated parameters that might have contributed to oral PrEP failures in groups 1 and 2 from our study. In group 1, we found that macaques acquiring virus in spite of daily TDF PrEP after the week 2 and 3 challenges had similar plasma TFV concentrations compared to animals that resisted infection during the initial 8-week period in this group (Figure 6A). The measured plasma TFV concentrations at week 2 (steady-state) in these animals were similar to plasma concentrations obtained 1 hour after a single dose of HOsm high douche (Supplemental Figure 6A) during our PK determinations. In fact, plasma TFV concentrations did not predict virus acquisition for group 1 during high- and reduced-dose TDF treatment (*r* = −0.2414, *P* = 0.4848) (Figure 6B). In addition, no statistical differences were noted in peak acute viremia (*P* = 0.40) for group 1 animals infected during high-dose and reduced-dose TDF daily treatment (Figure 6C). Analysis of plasma TFV and FTC concentrations from group 2 animals showed essentially the same absence of correlation between virus acquisition and plasma drug concentrations or peak viremias (Figure 6, D–H). The group 2 plasma TFV concentrations at challenge week 2 were similar to the group 1 at challenge week 2 and comparable to that of single-dose 1-hour PK of HOsm high douche (Supplemental Figure 6A). While tissue collections were not conducted during challenges, the PK data showed the most salient differences in TFV-DP to be noted in rectal tissue, for which HOsm douching achieved significantly higher drug concentrations than IOsm or steady-state PrEP at both 1 and 24 hours (Supplemental Figure 6, B and C).

## Discussion

Despite global, concerted efforts to control HIV among key populations, new infections among MSM have only modestly decreased for the past 2–3 years (1, 2, 46), suggesting that the anorectal route may fuel a significant part of new infections (47–49). The effectiveness of oral PrEP with TDF/FTC exceeds 90% if good adherence is maintained. Even intermittent PrEP is projected to deliver close to 90% protection, though a postcoital PrEP strategy has been reported to lead to only 40% adherence in a Ugandan cohort (50, 51). However, PrEP uptake and persistence are limited by perception of risk, antiretroviral drug (ARV) association with HIV stigma, limited-access areas (e.g., rural and Southern areas in the United States and resource-limited settings globally), and high cost among other variables (10, 52–54). Because the protective efficacy of daily oral PrEP is so dependent upon high levels of adherence, which is challenging to maintain, development of novel PrEP strategies has evolved in divergent directions toward (1) long-acting ARV delivery platforms that trade off the benefits of minimal need for sustained attention to PrEP adherence against continuous systemic drug exposure, even during periods without HIV acquisition risk and (2) on-demand methods that minimize systemic exposure and maximize adherence by medicating parasexual products commonly used prior to sex, e.g., sexual lubricants and douches. For example, given the common practice of rectal douching with an enema prior to or after RAI, a douche formulation presents a unique opportunity requiring little to no behavioral change compared with oral and other topical approaches (25, 29).

We have previously demonstrated in monkeys that TFV HOsm douche formulations can markedly promote local drug uptake and faster enzymatic turnover to TFV-DP in colorectal tissues compared with IOsm douche formulations (37). These studies also demonstrated protection of colorectal biopsies collected after douching in explant challenges, setting the stage for the in vivo challenge studies reported herein. However, there were several additional questions that we still needed to resolve. First, our HOsm delivery led to a relatively high peak of plasma TFV but relatively rapid elimination, and thus, we turned to other TFV prodrugs and formulations in attempts to prolong drug (TFV-DP) PK. TAF, a prodrug of TFV, has been shown to deliver the active metabolite to target cells more efficiently at lower doses, thereby reducing systemic exposure to TFV and the risk of renal toxicity (55). HIV-infected patients on oral TAF (25 mg) had 7-fold increased TFV-DP concentrations in peripheral blood mononuclear cells (PBMCs) compared with patients on oral TDF (300 mg) (56). In clinical trials, daily oral dosing of TAF, in fixed-dose combination with FTC, has proven noninferior to oral daily TDF and FTC in MSM and TGW. CMX157 is a lipid conjugate designed to remain intact in the blood and exploit natural lipid uptake pathways to achieve high intracellular TFV-DP. In vitro, CMX157 was reportedly 300-fold more active than the parent drug TFV against multiple viruses in several cell systems (57). We did determine systemic levels of TAF and CMX157 during PK studies, since our main focus was to test whether these prodrugs would provide PK benefits over TFV when formulated as HOsm douches.

A key limitation to TAF and CMX157 was solubility at higher concentrations (5.28 mg/mL, which led to the testing only of lower doses: 1.76 mg/mL). However, even when comparing low-dose TFV with the prodrugs TAF and CMX157 with each delivered topically in HOsm fluid, we found no apparent benefits over TFV, based on plasma TFV, colorectal tissue TFV-DP concentrations, or ex vivo protection. Plasma and colorectal TFV concentrations and tissue TFV-DP concentrations were the lowest overall with the CMX157 HOsm douche. One caveat may be that CMX157 may not be optimally metabolized by monkeys (Walid Heneine, Division of HIV Prevention, Centers for Disease Control and Prevention, personal communication), though in vitro side-by-side comparison of PBMCs from various nonhuman primate species did not support that notion in our hands (data not shown).

Based on the lack of benefit in using other prodrugs and douche formulations, we proceeded to compare the high-dose HOsm and IOsm TFV douche formulations with PrEP as a gold standard in a stringent efficacy study against repeat exposures to SHIV162p3. Of note, the same virus stock was used to challenge immunized monkeys intrarectally at a dose 4-fold more diluted than used here, which also infected all controls (58). Our challenge model was stringent enough to overcome oral PrEP for more than half of the animals. However, this challenge stringency also allowed for the determination of a superior protection efficacy of the HOsm high-dose TFV formulation. Of note, the absence of protection from the IOsm formulation clearly demonstrated that the enhanced uptake of drug into the tissues was associated with blocking the virus acquisition. In addition, the results further suggest that initial blockade of incoming virus at the portal of infection may be more effective than beyond, based on the similar TFV concentrations found in plasma between HOsm douche and PrEP and the much lower concentrations of systemic TFV in draining and peripheral lymphoid tissues compared with rectal mucosa (Supplemental Figure 2). While clearly the PK of this approach remains to be fully validated in humans, our results suggest that HOsm delivery of drugs has the potential to provide an alternative means of protection that does not require constant therapy.

Among the MSM who regularly douche prior to RAI, multiple douches applied in rapid succession rather than a single douche is far more common (79%), with 20% indicating 5 or more douches. To mimic clinical practice and understand both the PK and safety in macaques, we performed the repeated douche studies with varying douche volume. We observed no changes in colon histology, blood chemistry, or hematology after the 30 mL and 60 mL repeated douche sequences. The larger 60 mL volume resulted in a smaller percentage of retained douche volume,but a larger absolute volume. There appears to be a higher systemic exposure 1 hour after the repeated dose sequences and a markedly higher colon tissue TFV-DP concentration 24 hours after the 60 mL (5 times) sequence, but the very sparse sampling at only 1 and 24 hours after douching makes it challenging to estimate the temporal patterns between these points in time. Accumulation of colon tissue TFV-DP 24 hours after dosing without signs of toxicity could provide an extended period of protection if these data can be recapitulated in future studies. Several repeat-dose clinical studies are ongoing to examine the PK and safety of repeated douching with greater temporal granularity. Initially we conducted rapidly repeated douche administration with a 30 mL douche formulation given to the monkeys. The 30 mL dosing volume was selected from a range of volumes tested in previous studies with the aim to achieve the highest volume leading to minimal excretion of fluids over a 90-minute period in awake animals and ensure sufficient time for drug uptake. However, we found great variation in fluid retention in the anesthetized animals and therefore resorted to doubling the volume of douches to model fluid release. The main concern we wished to address, toxicity, did not materialize, attesting to the safety of the approach. As outlined above, though, the TFV-DP concentrations in colorectal tissues at 24 hours were unprecedented (Figure 2), suggesting the potential for extended protection, a notion that we still need to address.

The combination of findings in our macaque studies presented here — safety of single and multiple douche doses, high colon tissue drug uptake when compared with systemic drug exposure, protection of colon tissue explants ex vivo, and, most importantly, superior protection by a single on-demand, high-dose HOsm douche when compared with steady-state daily oral TDF/FTC dosing — clearly warrant further development of this on-demand, behaviorally congruent rectal TFV douche for PrEP. TFV is simpler to synthesize and more stable in solution than its prodrugs. Further, a pericoital douche formulation reduces the amount of drug used over time compared with daily oral PrEP strategies, reducing toxicity, cost, and need for daily adherence. While the clinical trials were initiated with a liquid formulation (125 mL), additional formulations will include a sachet version for ease of transport and use. Compared with long-acting strategies in development, our strategy reduces both systemic exposure and interactions with the health care system. In our model, our results demonstrate the ability to safely deliver a rapidly protective rectal microbicide formulation for PrEP worthy of clinical development of this on-demand, topical, and behaviorally congruent PrEP alternative.

## Methods

### Study animals.

Thirty-six healthy male adult Indian rhesus macaques (*Macaca mulatta*) naive for exposure to SIV or SHIV were used for this PK and efficacy study in 6 groups of 6 male rhesus macaques: body weight mean ± standard deviation 12.8 ± 0.5 kg; age 10.2 ± 0.4 years; and negative for SIV, simian retrovirus, and simian T lymphotropic virus. The animals were typed for Mamu A01, B08, and B17 to exclude animals expressing the last 2 alleles, while Mamu A01^+^ macaques were evenly distributed among the groups. All animals were housed and maintained at the New Iberia Research Center (NIRC) of the University of Louisiana at Lafayette in accordance with the rules and regulations of the Committee on the Care and Use of Laboratory Animal Resources. SHIV-infected macaques were humanely euthanized at the end of the study in accordance with the American Veterinary Medical Association Guidelines on Euthanasia, June 2007 (59).

### Douche formulations.

TFV (FT104801401), TDF (FT280311801), and FTC (NC056451801) were all sourced from Carbosynth. TAF (GS-7340; lot C11/019W) was provided by Gilead Sciences. CMX157 (CMX157 potassium salt; lot 021) was provided by Chimerix, Inc. (57). IOsm or HOsm low dose (1.76 mg/mL) and/or high dose (5.28 mg/mL) of TFV-based or TFV prodrug–based (TAF and CMX157) douche formulations were prepared as previously described (37, 43) at neutral pH. Briefly, 5.28 mg/mL TFV (high dose) was dissolved in purified water by addition of 0.6% (vol/vol) sodium hydroxide 18% (wt/vol) solution and 0.79% (wt/vol) sodium chloride (IOsm) or 0.33% (wt/vol) sodium chloride (HOsm). Similarly, 1.76 mg/mL TFV (low dose) was dissolved in purified water by addition of 0.2% (vol/vol) sodium hydroxide (18%, wt/vol) solution and 0.86% (wt/vol) sodium chloride (IOsm) or 0.41% (wt/vol) sodium chloride (HOsm). Due to the poor solubility of TAF in water (4.7 mg/mL) (55), only the low (1.76 mg/mL) dose of TAF was tested. For CMX157, the dose was adjusted to 3.72 mg/mL to match the TFV content in the low-dose HOsm TFV (1.76 mg/mL) formulation. All IOsm and/or HOsm douche formulations and oral TDF and TDF/FTC regimens are detailed in Figure 1.

### PK studies.

A crossover study design was used to evaluate single-dose douches (30 mL per monkey) for PK assessment of TFV- or TFV prodrug–based IOsm and/or HOsm formulations, as well as single dose of oral TDF with a human-equivalent high dose (22 mg/kg) or low dose (5 mg/kg) in groups of 6 rhesus macaques each, with a minimum of a 4-week washout period between administrations. In addition, 1-week oral daily steady-state PK of 22 mg/kg TDF alone or TDF (22 mg/kg) combined with 20 mg/kg FTC (represents a dose equivalent to human PrEP) was performed. Macaques underwent baseline blood collection, followed by a single 30 mL douche administration intrarectally (up to 10 cm) via a lubricated 8″ French catheter. The 30 mL volume was selected based on the volume retention of fluid administered intrarectally, with minimal excretion over a 150-minute period in awake animals, to ensure adequate time for drug uptake. Blood samples were collected at 1, 3, 6, 24, and 72 hours (for single-dose PK) or on days 5 and 7 (for the 1-week PK) postadministration and stored at –80°C for determination of TFV and TFV-DP concentrations. Colorectal biopsies (within 10–15 cm from the anal verge) were collected postdosing at 1, 24, and 72 hours or on day 7 using 3.2 mm biopsy forceps (Radial Jaw 4 Biopsy Forceps, Boston Scientific) for determining tissue drug concentration, isolating rectal CD4^+^ lymphocytes, assessing local tissue damage, or assessing protective efficacy ex vivo viral challenges of colorectal explants. Terminal PK was also assessed from multiple lymphoid tissues after euthanasia and sample collections at 3 or 24 hours after dosing for determination of systemic tissues’ drug levels.

### Sample analysis.

Systemic and compartmentalized TFV, FTC, TFV-DP, and FTC-TP concentrations were determined by liquid chromatographic-tandem mass spectrometry assays as described previously (60, 61). The lower limits of quantification (LLOQ) of TFV and FTC in plasma and homogenized colorectal tissue were 0.31 ng/mL and 0.05 ng/sample (TFV), and 0.31 ng/mL and 0.25 ng/sample (FTC), respectively. Tissue results were normalized to the weight of tissue analyzed and reported as ng/mg. TFV-DP and FTC-TP were quantified from homogenized tissue lysates, lymphoid lysates, or purified CD4^+^ cells isolated from colorectal tissue biopsies using a previously described indirect enzymatic approach (61, 62). The assay LLOQ for these determinations were 50 fmol/sample; metabolite results were normalized to cell counts or tissue weights. Assays were validated in accordance with FDA’s Guidance for Industry: Bioanalytical Method Validation recommendations (63). C_max_, T_max_, and AUCs from *t* = 0 through 24 hours (AUC_24_) or to the last measurable concentration (AUC_last_) were calculated using Phoenix WinNonLin v6.2 software.

### Ex vivo explant cultures.

Efficacy of selected TFV- or TAF-based douche formulations was assessed by assessing prevention and/or attenuation of virus replication by colorectal explants after ex vivo challenge in tissue culture. Colorectal biopsies were collected at 1, 24, and 72 hours after douching in groups of 6 rhesus macaques or from untreated control macaques. Colorectal biopsy explants were set in tissue culture media and challenged with high doses of SHIV162p3 and SIVmac251 ex vivo, excess input virus was washed out, and the kinetics of viral replication were assessed by the measurement of SIVgag p27 in culture supernatants collected on days 4, 7, and 10 as described previously (37).

### Efficacy study design.

Five intervention groups were compared as outlined in Supplemental Figure 1 comprising group 1 administered TDF orally daily, group 2 administered TDF/FTC orally daily, group 3 administered single TFV HOsm high-dose (5.28 mg/mL) douches prior to challenges, group 4 single TFV IOsm high-dose (5.28 mg/mL) douches before challenges, and group 5 HOsm or IOsm (*n* = 3 each) vehicle control douches. Intrarectal challenges were delivered once a week using 1,000 TCID_50_ SHIV162p3 per exposure. This SHIV162p3 virus stock was provided by Nancy Miller (Division of AIDS, National Institute of Allergy and Infectious Diseases, NIH). As outlined in Supplemental Figure 1, for the first series of 8 consecutive weekly SHIV challenges (1 to 8), group 1 and group 2 received oral daily TDF (22 mg/kg) alone, starting 25 hours prior to the initial exposure and TDF (22 mg/kg)/FTC (20 mg/kg), initiated 7 days before the initial exposure, respectively. Only the TDF/FTC regimen could achieve steady-state concentrations by the time of the initial SHIV challenge. For the second series of 8 consecutive weekly SHIV challenges, daily doses of TDF/FTC were decreased to 5 mg/kg of TDF and 5 mg/kg FTC. Subsequent challenges in animals resisting the 16 challenges were done in the absence of treatment to ascertain all animals’ susceptibility to mucosal infection. For groups 3–5, the first series of weekly challenges were conducted 1 hour after douching. Uninfected animals underwent a second round of 8 consecutive challenges administered 24 hours after douching, and the animals that resisted 16 challenges (8 challenges 1 hour after douching and 8 challenges 24 hours after douching) underwent a third round of 8 challenges administered 48 hours after douching. Animals confirmed as infected via positive plasma viral loads had further treatments and challenges discontinued. Plasma viral loads were quantified by SIVgag-specific quantitative PCR at NIRC and with a detection limit of 100 viral RNA copies/mL.

### PK and safety of repeated short-term intrarectal douches.

The anesthetized macaques were placed in recumbency and administered 30 mL or 60 mL TFV HOsm high dose douche (5.28 mg/mL) intrarectally using a lubricated 8″ French catheter. After 5 minutes, the animals were placed on their back and the abdomen was manually pressed in a downward motion 3 times to try to expel colorectal fluid before administering the next douche; the sequence was repeated for a total of 5 consecutive TFV douches within 25 minutes. The ejected fluid volumes were recorded after each procedure. Blood and 1 colorectal biopsy were collected right after the last administration (postdose) 0, 1, and 24 hours, respectively, and stored at –80°C for determination of TFV and TFV-DP concentrations. To assess local tissue tolerability after repeated high-dose TFV douching, colorectal biopsies were collected from 5 colorectal sites 1 week before and at 24 hours after each dose time point. Biopsy specimens were either flash-frozen for TFV and TFV-DP determination or immediately fixed in 4% paraformaldehyde followed by passage in graded ethanol and paraffin embedding, sectioned, and stained with hematoxylin and eosin. Histological evaluations were performed by an American College of Veterinary Pathologists board-certified pathologist. In addition, the animals were monitored for systemic toxicity by complete blood counts and a complete serum chemistry panel before and at 24 and 72 hours after the last dose.

### Statistics.

Protection efficacy was analyzed using the Cox proportional hazard model in SPSS Statistics software to estimate the relative HR (95% CI), for the per-exposure relative reductions of acquisition risk of the treatment and control groups, considering the number of challenges as the time variable. Graphical methods of model assessment supported the use of Cox proportional hazards regression. The cumulative survival rates of uninfected macaques were plotted as Kaplan-Meier curves and were compared by a log-rank test. Two-group comparisons were evaluated by the exact Wilcoxon rank-sum test. The 1-way ANOVA or Kruskal-Wallis test was used for comparisons across 3 or more groups. The Spearman rank correlation test was used to assess the relationships between pairs of parameters. All statistical analyses were considered significant if they produced *P* values of < 0.05.

### Study approval.

The entire study (protocol 2016-8799-068) was reviewed and approved by the University of Louisiana at Lafayette IACUC. All animals were housed and maintained at the NIRC of the University of Louisiana at Lafayette in accordance with the rules and regulations of the Committee on the Care and Use of Laboratory Animals. The NIRC facilities are fully accredited by the Association for Assessment and Accreditation of Laboratory Animal Care International and licensed by the US Department of Agriculture.

## Author contributions

PX, SG, MAM, JH, LME, LCR, RC, EJF, CWH, and FV designed the study. PX, SG, MAM, TH, RM, AAD, LW, and FV conducted the experiments. PX, SG, MAM, LME, LCR, RC, EJF, CWH, and FV analyzed the data. PX, SG, MAM, LME, EJF, CWH, and FV wrote the manuscript.

## Supplementary Material

Supplemental data

## Figures and Tables

**Table 1:**
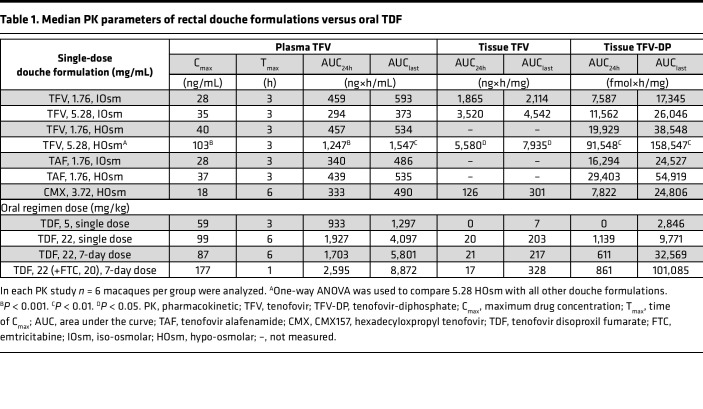
Median PK parameters of rectal douche formulations versus oral TDF

**Table 2:**
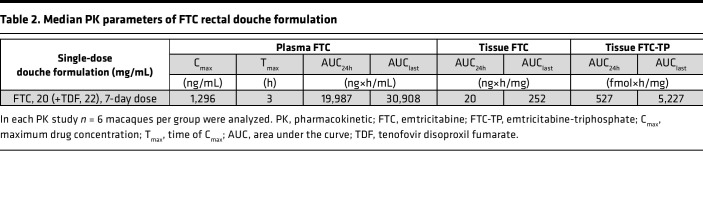
Median PK parameters of FTC rectal douche formulation

**Table 3:**
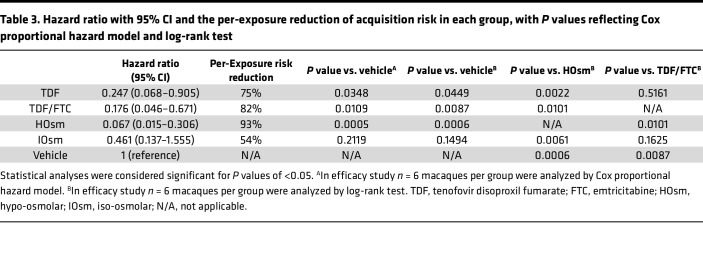
Hazard ratio with 95% CI and the per-exposure reduction of acquisition risk in each group, with *P* values reflecting Cox proportional hazard model and log-rank test

## Version 1 12/08/2022

Electronic publication
