# Supplementary material for: Hypo-osmolar rectal douche tenofovir formulation prevents simian/human immunodeficiency virus acquisition in macaques
Source: JCI Insight. 2022 Dec 8;7(23):e161577. doi: 10.1172/jci.insight.161577 (PMC9746910; doi:10.1172/jci.insight.161577)
Supplement: Supplemental data [file jciinsight-7-161577-s010.pdf]

Supplemental Materials for:

Hypo-osmolar Rectal Douche Tenofovir (TFV) Formulation Prevents SHIV Acquisition in Macaques

Peng Xiao[1], Sanjeev Gumber[2], Mark A. Marzinke[3,4], Thuy Hoang[5,8], Rohan Myers[1], Abhijit A. Date[5,10], Justin Hanes[5,9], Laura M. Ensign[5-9], Lin Wang[11-13], Lisa C. Rohan[11-13], Richard Cone[3], Edward J. Fuchs[3], Craig W. Hendrix[3,5,8], Francois Villinger[1]*

[1]New Iberia Research Center, University of Louisiana at Lafayette, New Iberia, LA

[2]Division of Pathology, Yerkes National Primate Research Center, Emory University, Atlanta, GA

[3]Departments of Medicine (Clinical Pharmacology), and [4]Pathology, Johns Hopkins University School of Medicine, Baltimore, MD

[5]Center for Nanomedicine, Departments of [6]Ophthalmology, [7]Chemical & Biomolecular Engineering, [8]Pharmacology and Molecular Sciences, [9]Biomedical Engineering, Johns Hopkins University, Baltimore, MA

[10]Department of Pharmacology and Toxicology, R. K. Coit College of Pharmacy, Department of Ophthalmology and Vision Science, University of Arizona, Tucson, AZ 85715

[11]Department of Pharmaceutical Sciences, School of Pharmacy, [12]Department of Obstetrics, Gynecology and Reproductive Sciences, University of Pittsburgh, and [13]Magee-Womens Research Institute, Pittsburgh, PA

*Address correspondence to:

Francois Villinger
New Iberia Research Center
University of Louisiana at Lafayette
4401 W Admiral Doyle Drive
New Iberia, LA 70560, USA
Phone: 337-482-0225
Email: Fjv5939@louisiana.edu

Supplemental Figure 1

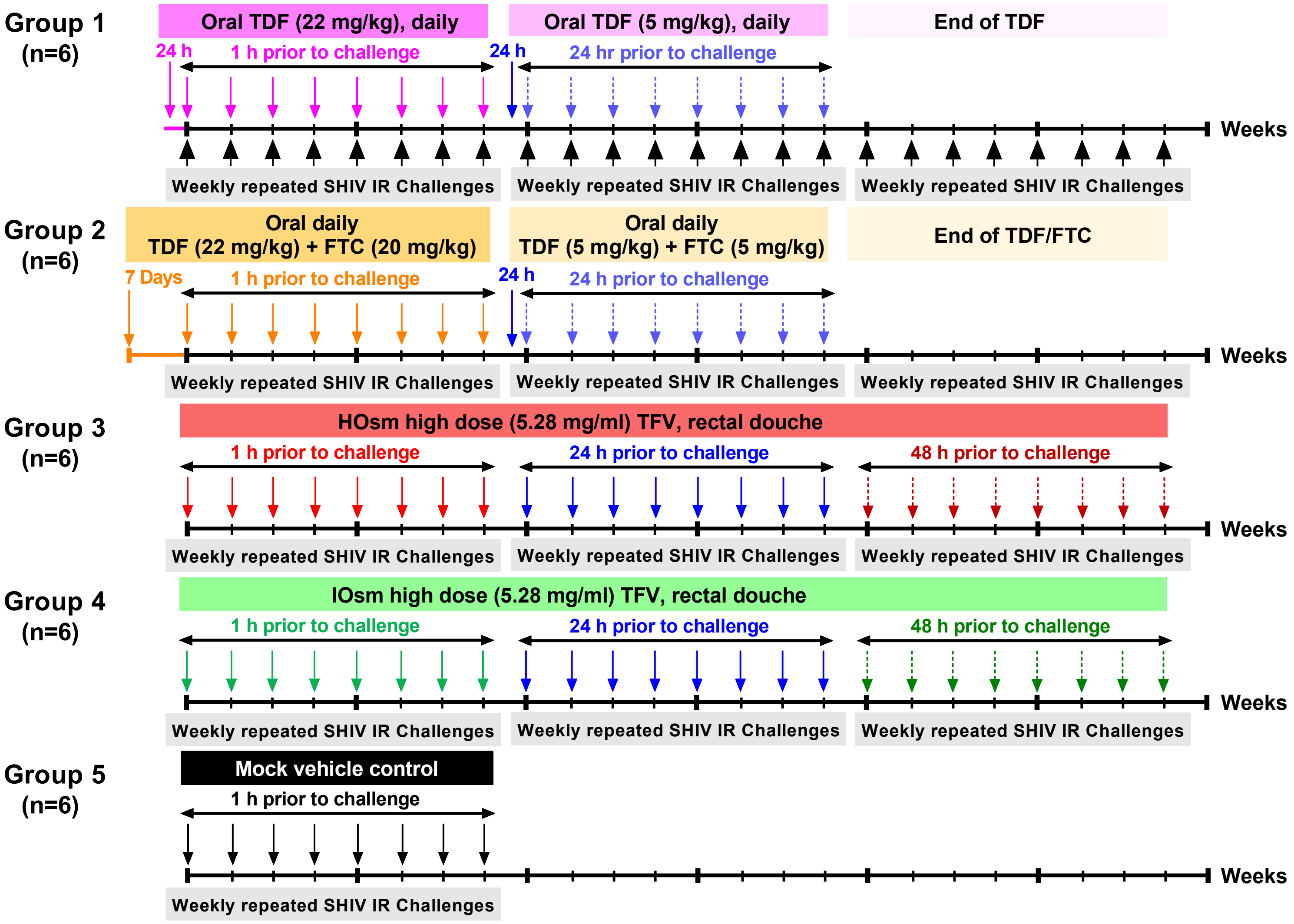

Supplemental Figure 1. Schematic diagram of five interventions groups in efficacy trail. For the first series of 8 consecutive weekly SHIV162p3 (TCID50=1000) intrarectal challenges, Group 1 (n=6) was treated daily with oral TDF (22 mg/kg) starting one day before the initial SHIV challenge. Group 2 (n=6) received daily oral TDF (22 mg/kg)/FTC (20 mg/kg), starting 7 days before the initial SHIV exposure. Animals in these 2 groups that remained uninfected after 8 SHIV exposures, had their daily drug doses reduced to 5 mg/kg TDF (group 1) or 5 mg each of TDF and FTC (group 2) for the second series of 8 SHIV exposures. Groups 1-2 animals that resisted 16 SHIV exposures were further challenged in the absence of oral drug treatment. Group 3 (n=6) was administered single high dose (5.28 mg/ml) HOsm TFV rectal douche 1 hour prior to SHIV exposure and Group 4 (n=6) was administered single high dose (5.28 mg/ml) IOsm TFV rectal douche 1 hour prior to SHIV exposure for 8 consecutive challenges. Group 5 (n=6) was mock control administered either HOsm (n=3) or IOsm (n=3) vehicle rectal douche 1 hour prior to SHIV exposure. Next, groups 3 and 4 animals that resisted infection underwent a second round of 8 consecutive weekly SHIV162p3 rectal challenges, following a single dose of each douche formulation administered 24 hours prior to each rectal SHIV exposure. Finally, groups 3-4 uninfected animals underwent a third round of challenges administered 48 hours post douching. Each minor bar on the x-axis represents a week. TDF: tenofovir disoproxil fumarate; FTC: emtricitabine; TFV, tenofovir; HOsm: hypo-osmolar; IOsm: iso-osmolar.

Supplemental Figure 2

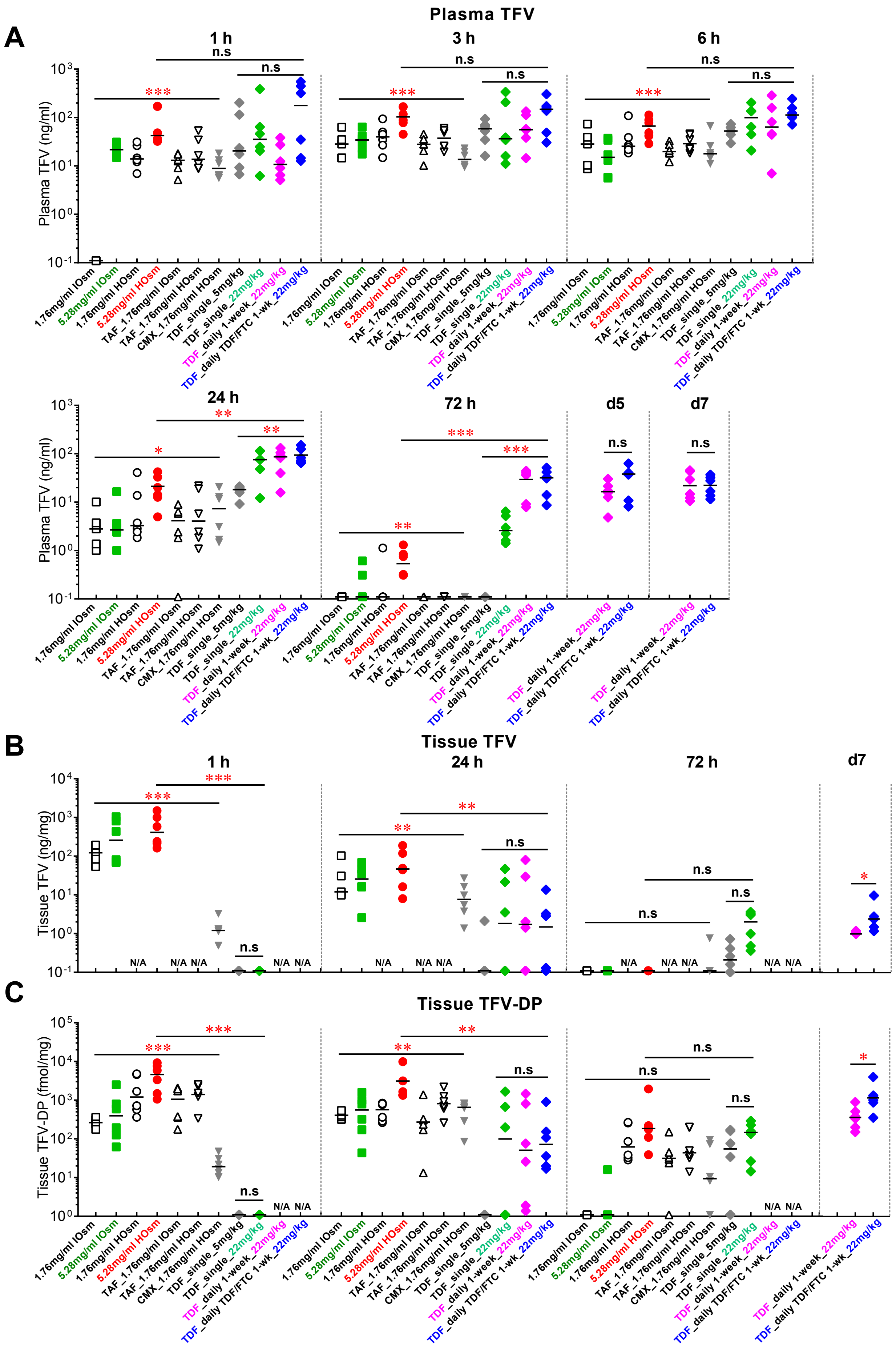

Supplemental Figure 2

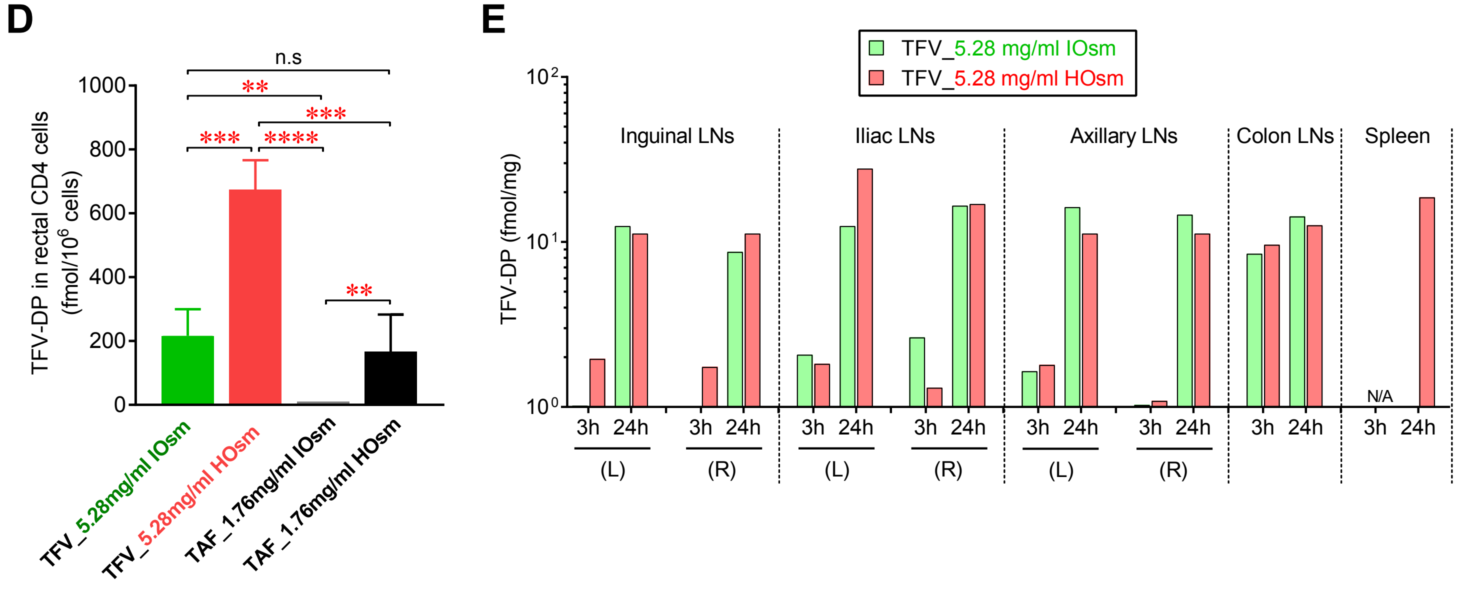

Supplemental Figure 2. Pharmacokinetic assessments following IOsm and HOsm of TFV or TFV prodrug formulated douches and oral TDF and TDF/FTC regimens in groups of 6 monkeys each after administration at indicated time points. (A) plasma TFV, (B) colorectal tissue TFV, (C) rectal tissue TFV-DP concentrations, (D) rectal CD4+ cells TFV-DP concentrations at 1 hour and (E) TFV-DP levels in inguinal, iliac, axillary and colon draining lymph nodes (LNs) at 3 hours or 24 hours post douche administration. N/A, not measured. Error bars indicate the standard errors of the means. Two-group comparisons were evaluated by the exact Wilcoxon rank sum test. The One-way ANOVA was used for comparisons across three or more groups. The level of significance is indicated by *P*-values: **P* <0.05; ***P* <0.01; *** *P* <0.001; **** *P* <0.0001; n.s, *P* >0.05, not significant. TFV: tenofovir; TFV-DP: tenofovir-diphosphate; TAF: tenofovir alafenamide; CMX157: hexadecyloxpropyl tenofovir; TDF: tenofovir disoproxil fumarate; FTC: emtricitabine; IOsm: iso-osmolar; HOsm: hypo-osmolar.

Supplemental Figure 3

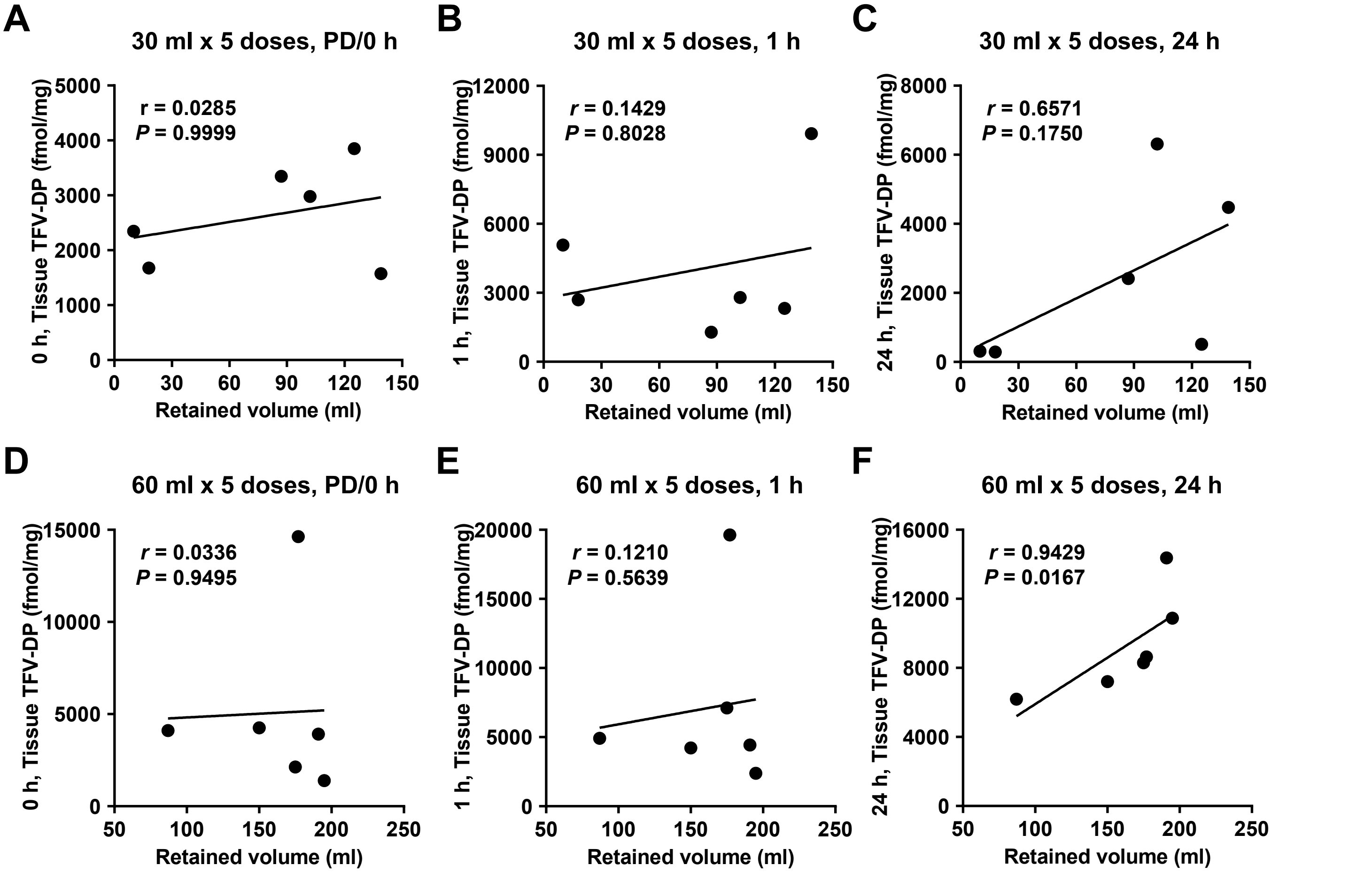

Supplemental Figure 3. Pharmacokinetic correlation of tissue TFV-DP concentration with retained volumes after 5 repeated short-term intrarectal douches of TFV HOsm high formulation. Analyses were conducted after 5 consecutive (every 5 minutes) rectal administrations of 30 ml in group of 6 monkeys at (A) post-dose (0 hour), (B) 1 hour, and (C) 24 hours; or after 5 consecutive (every 5 minutes) rectal administrations of 60 ml in group of 6 monkeys at (D) post-dose (0 hour), (E) 1 hour, and (F) 24 hours, respectively. The correlation coefficients (r) and P values were derived using Spearman rank analysis. Statistical analyses were considered significant of *P* values of <0.05. TFV: tenofovir; TFV-DP: tenofovir-diphosphate; PD: post-dose after 5 consecutive rectal douches.

Supplemental Figure 4

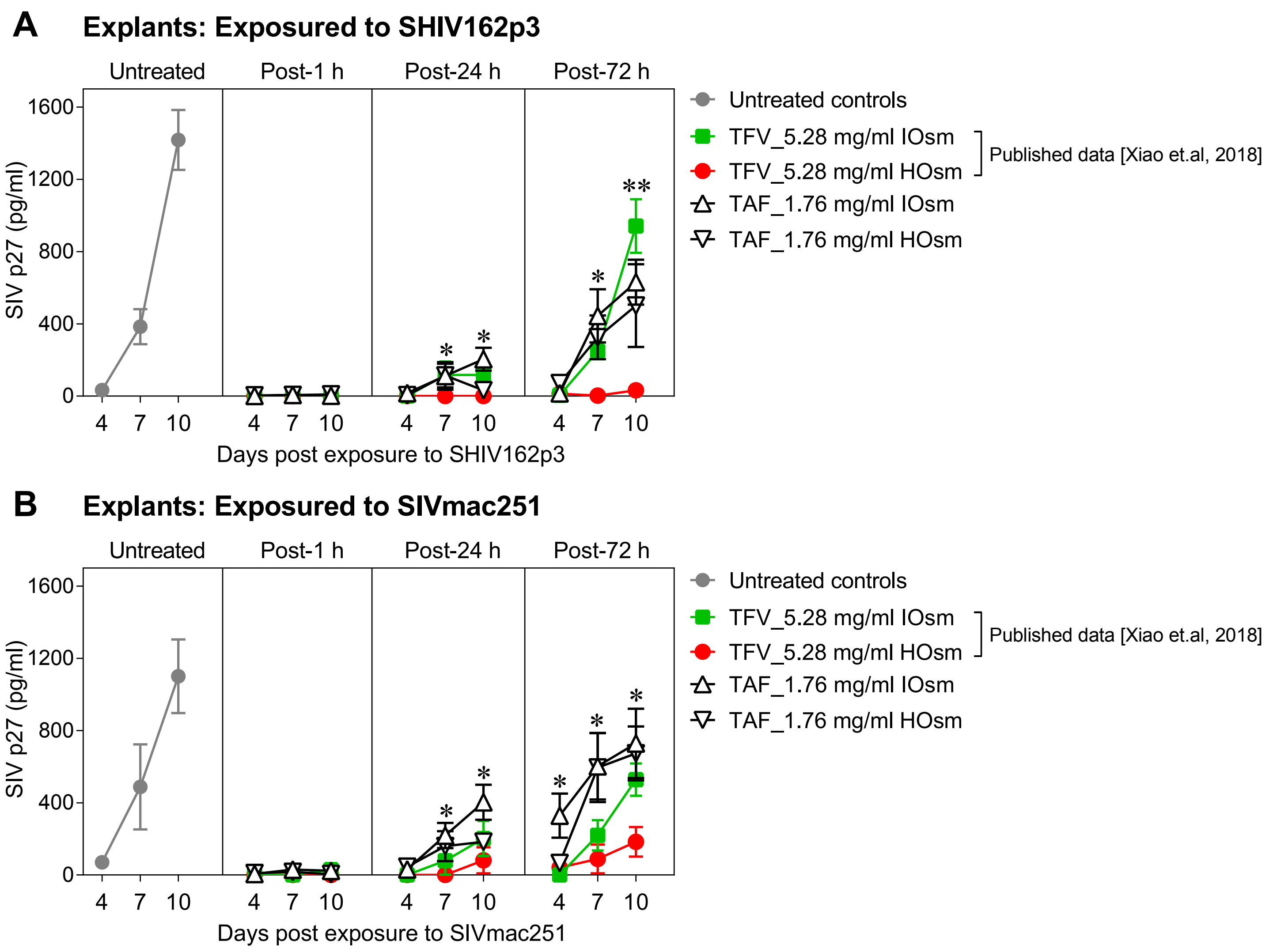

Supplemental Figure 4. Activities of four douche formulations against (A) SHIV162p3 or (B) SIVmac251 infection of *ex vivo* colorectal tissue explants collected at 1, 24 and 72 hours post administration. Six macaques in each formulation group were tested. Untreated macaques (n=3) were used as control. Freshly collected colorectal explants (4 biopsies/animal) were exposed to either SHIV (2 biopsies) or SIV (2 biopsies) viruses for 2 hours followed by washing and culturing in 500 µl explant culture medium. On days 4, 7 and 10, the supernatants were harvested for SIVgag p27 measurement by ELISA. Error bars reflect the standard error of the mean. The level of significance is indicated by *P*-values: *$P < 0.05$; ** $P < 0.01$. TFV: tenofovir; TAF: tenofovir alafenamide; IOsm: iso-osmolar; HOsm: hypo-osmolar.

Supplemental Figure 5

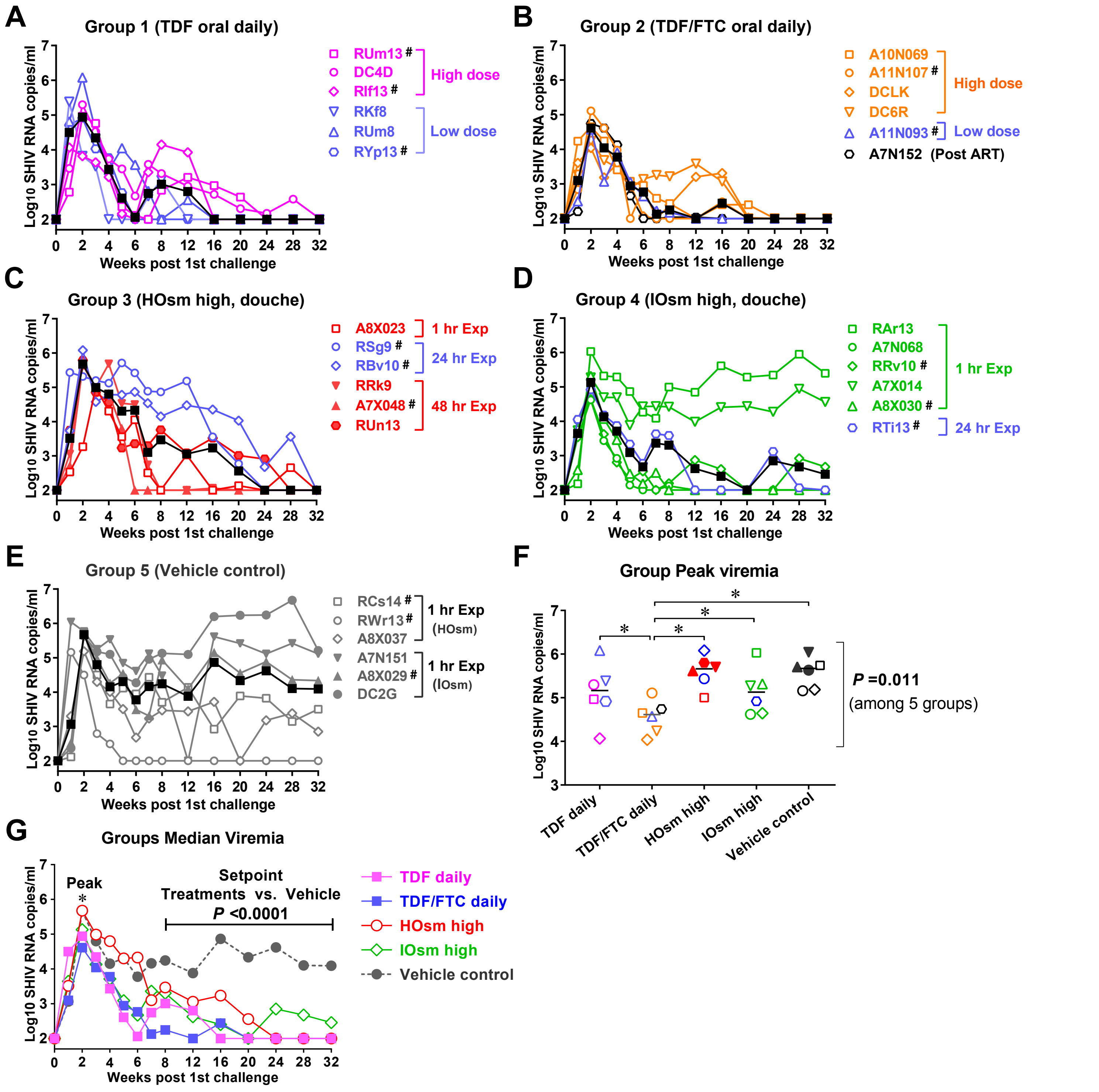

Supplemental Figure 5. Viral loads post-infection. Panels (A) to (E) illustrate individual plasma viral load kinetics post-infection in Group 1 (n=6) oral daily TDF; Group 2 (n=6) TDF/FTC oral daily; Group 3 (n=6) administered single HOsm high dose (5.28 mg/ml) TFV rectal douche; Group 4 (n=6) administered single IOsm high dose (5.28 mg/ml) TFV rectal douche, and Group 5 (n=6) administered control vehicle douche. The symbol # indicates Mamu A01-positive macaques distribution among five groups. (F) Difference among the five groups in plasma peak viral loads was evaluated using the Kruskall-Wallis test. The pairwise Wilcoxon test was used to compare Group 2 (TDF/FTC oral daily) to each of the others. The level of significance is indicated by *P*-values as follows: **P* <0.05. (G) Median plasma viremia post-infection by groups. The median chronic viral load setpoint (from week 8 to week 32 post infection) were compared between treatments (Groups 1-4) and vehicle control (Group 5). TDF: tenofovir disoproxil fumarate; FTC: emtricitabine; TFV: tenofovir; HOsm: hypo-osmolar; IOsm: iso-osmolar.

Supplemental Figure 6

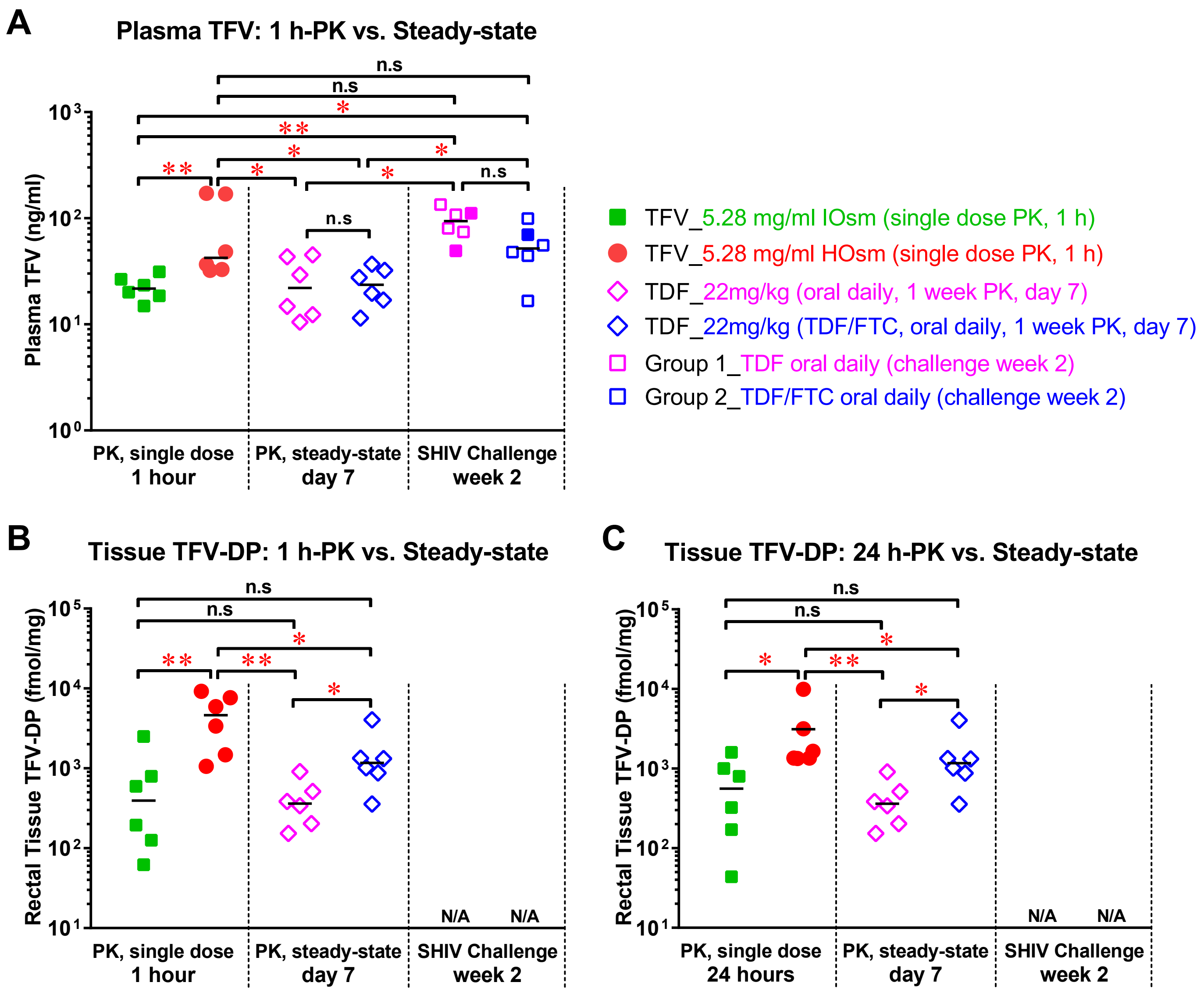

Supplemental Figure 6. (A) Comparison of plasma TFV concentrations among post-1 hour single high dose PK of IOsm and HOsm douche (1 h-PK), one-week oral daily PrEP PK (steady-state on day 7) and two oral daily PrEP groups in efficacy trial (Plasma samples from monkeys undergoing SHIV challenge week 2, Figure 6). Comparison of tissue TFV-DP concentrations between single high dose douche PK at (B) post-1 hour (1 h-PK), and (C) post-24 hours (24 h-PK) with one-week oral daily PrEP PK (steady-state). To avoid micro-abrasions of mucosa that could facilitate virus transmission and infection, rectal tissue samples were not collected during challenges, thus tissue TFV-DP data were not available (N/A) from two oral PrEP groups in efficacy trial at week 2. In each PK study or efficacy trial n = 6 macaques per group were analyzed. The level of significance is indicated by *P*-values as follows: *P <0.05; ** P <0.01; n.s, P >0.05, not significant. TFV: tenofovir; TFV-DP: tenofovir-diphosphate; TDF: tenofovir disoproxil fumarate; FTC: emtricitabine; IOsm: iso-osmolar; HOsm: hypo-osmolar.
